# Identifying outcome-based indicators and developing a curriculum for a continuing medical education programme on rational prescribing using a modified Delphi process

**DOI:** 10.1186/1472-6920-8-33

**Published:** 2008-05-30

**Authors:** Hamideh M Esmaily, Carl Savage, Rezagoli Vahidi, Abolghasem Amini, Mohammad Hossein Zarrintan, Rolf Wahlstrom

**Affiliations:** 1 Division of International Health (IHCAR), Department of Public Health Sciences, Karolinska Institutet, Stockholm, Sweden; 2 Educational Development Center (EDC), Tabriz University of Medical Sciences, Tabriz, Iran; 3 National Public Health Management Center (NPMC), Tabriz, Iran; 4 PhD student, Department of Learning, Informatics, Management and Ethics (LIME), Karolinska Institutet, Stockholm, Sweden; 5 Health and Nutrition Department, Tabriz University of Medical Sciences, Tabriz, Iran; 6 Medicine Department, Tabriz University of Medical Sciences, Tabriz, Iran; 7 Pharmacy Department, Tabriz University of Medical Sciences, Tabriz, Iran; 8 Department of Public Health and Caring Sciences, Family Medicine and Epidemiology, Uppsala University, Uppsala, Sweden

## Abstract

**Background:**

Continuing medical education (CME) is compulsory for physicians in Iran. Recent studies in Iran show that modifications of CME elements are necessary to improve the effectiveness of the educational programmes. Other studies point to an inappropriate, even irrational drug prescribing. Based on a needs assessment study regarding CME for general physicians in the East Azerbaijan province in Iran, rational prescribing practice was recognized as a high priority issue. Considering different educational methods, outcome-based education has been proposed as a suitable approach for CME. The purpose of the study was to obtain experts' consensus about appropriate educational outcomes of rational prescribing for general physicians in CME and developing curricular contents for this education.

**Methods:**

The study consisted of two phases: The first phase was conducted using a two-round Delphi consensus process to identify the outcome-based educational indicators regarding rational prescribing for general physicians in primary care (GPs). In the second phase the agreed indicators were submitted to panels of experts for assessment and determination of content for a CME program in the field.

**Results:**

Twenty one learning outcomes were identified through a modified Delphi process. The indicators were used by the panels of experts and six educational topics were determined for the CME programme and the curricular content of each was defined. The topics were 1) Principles of prescription writing, 2) Adverse drug reactions, 3) Drug interactions, 4) Injections, 5) Antibiotic therapy, and 6) Anti-inflammatory agents therapy. One of the topics was not directly related to any outcome, raising a question about the need for a discussion on constructive alignment.

**Conclusions:**

Consensus on learning outcomes was achieved and an educational guideline was designed. Before suggesting widespread use in the country the educational package should be tested in the CME context.

## Background

The need for continuing medical education (CME) as a part of a medical doctor's professional development has been recognized all over the world [1,2]. Since 1991, CME has been compulsory for all Iranian physicians and other health professionals.

Traditionally, general physicians working in primary care as general practitioners (here referred to as GPs) have obtained their CME through lectures and written materials. These methods may increase their knowledge, but there is no clear evidence that their skills or performance are impacted [3-9].

Although outcome-based education (OBE) initially was proposed for undergraduate medical education and training [10], it is now increasingly recognized as a useful approach for CME [11]. OBE can influence the entire process of education, i.e. decisions about the content, formulation of aims, educational strategies, the teaching methods, the assessment procedures and the educational environment [10].

Several advantages have been suggested for adopting an OBE model for medical education, such as helping to increase the relevance of the education to the future practice of medicine. OBE allows for a wide degree of participation in the curriculum design process as well as flexibility regarding the choice of educational strategies used. OBE appears to be acceptable to most teachers probably because the concepts of OBE are clear, easily understandable, and provide a robust framework for the curriculum [10,12].

Recent studies of CME for physicians in Iran show that modifications of CME elements are necessary to improve the effectiveness of the educational programs [13,14]. There are also convincing data showing excessive prescribing behaviour in Iran among GPs [15]. Some studies point to an inappropriate, even irrational drug prescribing [16,17]. According to an unpublished needs assessment study about CME for GPs in the East Azerbaijan province in Iran (2002), rational prescribing practice was recognized as a high priority issue.

Given the timeliness and importance of this issue in Iran, we are conducting a project aimed at evaluating the effectiveness of using OBE on GPs knowledge, skills, attitudes, and performance in a CME program on rational prescribing. No studies on OBE indicators for rational prescribing were found in the literature. The aim of this study was, therefore, to identify the appropriate outcomes and indicators for rational prescribing based on expert opinions and developing the curricular content in a CME program for GPs.

## Methods

The study was conducted between October 2005 and July 2006 in three phases. The first phase consisted of two rounds of a Delphi process. In the second phase, panel discussion sessions were used to further clarify, reach consensus, and then prioritize the learning outcomes and indicators. Finally, an appointed team determined content for the CME programme.

The Delphi technique was originally conceived of as a way to obtain the opinion of experts without necessarily bringing them together face to face [18]. This is what attracted us to the method. For our study, we used a modified version of the technique which also involved panel discussions with experts in the field of rational prescribing and CME. In the first two phases, the methodology was similar to what has been described previously [12,19].

A questionnaire was designed consisting of potential learning outcomes identified from a range of sources: WHO (World Health Organization) prescribing indicators [20,21], relevant documents [16,17,22,23], and topics covered by CME programs on prescribing in Iran. A small group of experts (a pharmacologist, a pharmacist, and two medical specialists) assisted the research team in defining a preliminary list of 16 potential outcomes with appropriate measurement indicators for each one.

### Participants

We selected 30 stakeholders who had a background from at least one of the following categories (some represented several categories): 1) seven experienced GPs, 2) four CME decision makers from Iranian medical science universities with a background in pharmacy, pharmacology, or health management and who work for the Ministry of Health and Medical Education (MOHME), 3) nine pharmacists, 4) seven pharmacologists, 5) six medical specialists. With the exception of the experienced GPs, all the participants were faculty members of four of the Universities of Medical Sciences in Iran as well as being CME trainers.

### Delphi process and panel discussion

The questionnaire, with the 16 potential outcomes including a definition of each outcome (to avoid any misunderstanding), was sent out to the participants. The respondents were asked if there was a need to include the outcome in a programme on rational prescribing. They answered using a 5-point Likert scale (1 = totally agree, 2 = partly agree, 3 = uncertain, 4 = partly disagree and 5 = totally disagree). They were also asked to suggest any other potential learning outcomes, based on their expertise in the area.

The results of the returned questionnaires were assessed by the research team and a second questionnaire was developed for the next stage of the Delphi process. It consisted of the initial potential learning outcomes from round 1 and ten new learning outcomes that had been suggested by the experts. In this round, each member of the study was asked to determine which of the proposed outcomes should be included by using a 4-point scale (1 = totally agree, 2 = partly agree, 3 = partly disagree and 4 = totally disagree) to avoid uncertain answers.

The questionnaire responses were coded and frequencies were determined using Microsoft Excel 2003 [24]. The results of the second stage of the Delphi process were submitted to a specially assigned panel of experts comprised of four CME decision-makers, four pharmacists or pharmacologists, four medical specialists, three GPs, one epidemiologist, and the first author. The task of the panel was to reach consensus on the final educational outcomes and indicators and to identify a suitable team of experts to develop the content.

### Developing the content

The team responsible for content development was comprised of seven experienced CME trainers, all who had participated in the panel discussion, and the first author. They identified educational topics for an outcome-based CME program in rational prescribing informed by the list of outcomes and outcome indicators that were developed from the second Delphi round. To each of these topics a small group of experts and CME trainers was assigned, with the task of developing the curricular content for that topic.

The results from each of the small groups were distributed to all the other teams one week prior to a final review meeting. At this meeting, ideas about the curricular content were discussed and consensus was reached after a revision of some of the topics. The final curricular content formed the basis for the OBE educational program that was to be used in the CME program on Rational Prescribing.

Ethical approval for the study was received from the Ethical Vetting Committee of the Iranian Ministry of Health and Medical Education. Informed consent was given through agreeing to participate in the different phases of the study.

## Results

### The Delphi process

Completed questionnaires were returned from 21 of the 30 participants in the first round. Nine of the participants (four medical specialists, two pharmacologists and three pharmacists) did not answer, despite the reminder we sent out one month later. These non-responders were contacted by e-mail and/or telephone; six replied that they were too busy to answer and three did not wish to participate as they saw no personal benefit.

Ten new potential outcomes were added in the second round based on the experts' suggestions. The second questionnaire was sent to the 21 experts who had answered in the first round. All but one responded with new ratings. Table 1 displays both the original outcomes and the ones suggested by the participants in round 1. Some suggestions were deemed to be beyond the scope of the programme (such as the importance of using a computerized prescription system to remedy prescribing errors and the importance of the influence of socio-economic factors on rational prescribing) or in conflict with course aims (for instance, criticisms about using the generic names of drugs) and therefore excluded from the process.

**Table 1:** Potential learning outcomes as assessed through the Delphi process

**Potential outcomes for Rational Prescribing (round 1)**	Agreement* (n = 21)	Agreement* (n = 20)
*Upon completion of the course, a doctor will be able to write a prescription that includes or considers:*	**Round 1**	**Round 2**
		
1. Date of the prescription	21	20
2. Name and age of the patient	21	20
3. Name and identification number of Iranian Medical Council of the prescriber**	21	20
4. Main complaint of the patient	11	16
5. Legible hand writing	20	20
6. Generic name of the drugs	16	18
7. Administration form of drugs	20	20
8. Strength of the drugs and dose frequency	20	20
9. Adequate duration of treatment	16	20
10. Latin abbreviation of terminology in drug use order	12	17
11. Appropriate number of drugs	16	20
12. To consider homogeneity of prescription per individual (all drugs prescribed pertain to the same individual)	18	19
13. Not prescribing drugs with the same pharmacological effect	14	19
14. Not prescribing drugs which have negative interactions with each other	17	19
15. Appropriate number and amount of antibiotics	19	20
16. Appropriate number of injections	17	20
		
**Suggested potential outcomes (added in round 2)**		
1. Contact telephone number of the prescriber		17
2. Refill information		18
3. Initial diagnosis		15
4. Time and manner of drug use		17
5. Necessary precautions		16
6. Necessary notifications about signals to continue or stop drug use		13
7. Notification about side effects of drugs in the prescription		6
8. Name of the foods which have negative interactions on drug efficacy and the treatment process		8
9. Appropriate no. of NSAID (Non-steroidal anti-inflammatory) drugs		20
10. Appropriate number of vitamins		19

* Sum of the number of respondents who answered "partly agree" and "totally agree".

** Compulsory in Iran

### The panel discussion

The panellists finalised the learning outcomes during two meetings based on the results of the Delphi process. They agreed to accept all outcomes which at least four out of five experts had agreed to. However, two outcomes ("Writing main complaint of the patient in the prescription" and "Appropriate number of vitamins") were eliminated and a part of the last outcome was expanded to include corticosteroids. Some of the experts were critical about using generic names because of the specific opinions that both doctors and patients can have regarding different brands. However, citing the WHO, it was suggested that this can be solved by adding the trade name after the generic name if there is a particular reason to prescribe a specific brand [21]. At the end of the second meeting, 21 learning outcomes and appropriate assessment indicators had been identified (Table 2).

**Table 2:** Rational prescribing outcomes and indicators for GP's CME programmes as agreed by an expert panel

**Outcomes**	**Assessment Indicator**
*Upon completion of the course, a participant should be able to:*	
1. Write the date of the prescription	1. Date of the prescription
2. Write the name of the patient	2. Name of the patient
3. Write the age of the patient	3. Age of the patient
4. Write the name and identification number of the Iranian Medical Council for the prescriber	4. Name and identification number of Iranian Medical Council of the prescriber
5. Write the contact telephone number of prescriber	5. Contact telephone number of prescriber
6. Write refill information	6. Refill information
7. Write the prescription clearly	7. Legible hand writing
8. Write the generic name of the drugs	8. Generic names of drugs
9. Write the administration form of the drugs	9. Administration form of drugs
10. Write the strength of the drugs (dose and dose frequency)	10. Strength of the drugs
11. Write the duration of treatment	11. Treatment duration
12. Use the Latin abbreviation of terminology in drug use order	12. Use Latin abbreviation terminology
13. Write the time and manner of drug use	13. Time and manner of drug use
14. Write the necessary precautions	14. Necessary precautions
15. Prescribe the appropriate number of drugs	15. Appropriate number of drugs
16. Consider homogeneity of prescription per individual (all drugs prescribed pertain to the same individual)	16. Homogeneity of prescriptions
17. Avoid prescribing drugs with the same pharmacological effect	17. Number of drugs in the same pharmacological group
18. Avoid prescribing drugs which have negative interactions with each other	18. Number of interactive drugs per prescription
19. Prescribe the appropriate number and amount of antibiotics	19. Number and amount of antibiotics per prescription and proportion of antibiotics prescribed
20. Prescribe the appropriate number of injections	20. Number of injections per prescription and proportion of injections prescribed
21. Prescribe the appropriate number of Anti-Inflammatory Agents [Corticosteroids and Non-steroidal anti-inflammatory drugs (NSAID)]	21. Appropriate number of Anti-Inflammatory Agents [Corticosteroids and Non-steroidal anti-inflammatory drugs (NSAID)]

### Content development

In the third phase, the team responsible for content development divided the 21 learning outcomes into six main areas for the educational programme: 1) Principles of prescription writing, 2) Adverse drug reactions (ADR), 3) Drug interactions, 4) Injections, 5) Antibiotic therapy, and 6) Anti-inflammatory agents therapy

The content for each of these six topics was then developed individually by six small teams of professionals who were experts in each topic. The curricula were distributed and validated by all participants in the final meeting, during which the content was finalized (Table 3).

**Table 3:** Curricular content for the programme on rational prescribing as developed by teams of CME trainers.

**Topics for the rational prescribing curriculum**	
1-Principles of prescription writing (1–16)*	**5-Antibiotic therapy (19)**
History of prescription writing	Value of taking culture samples for infections
Classification of drugs	Assessment of infectious organisms
Definition and format	Importance of host factors in selection of
Elements of prescription writing	antibiotics
Measurements	Adherence to correct indications
Mistakes and errors in prescription writing	Selection of antibacterial drug(s)
Abbreviations	Important factors for choosing form, dose and
Poor prescriptions	course of antibiotics
Rational prescriptions	Importance of switching antibiotics based on
**2-Adverse reactions to drugs**	culture and antibiogram results
Background	Pharmacology of antibiotic groups:
Epidemiology	○ Betalactamases
Etiology	○ Tetracyclines
Exaggeration of an intended pharmacologic	○ Aminoglycosides
action of the drugs	○ Macrolides
Toxicity unrelated to a drug's primary	○ Fluoroquinolones
pharmacological activity:	○ Sulfonamides
- Cytotoxic Reactions	**6- Anti-inflammatory agents therapy (21)**
- Immunologic Mechanisms	A) Corticosteroids
Diagnosis and treatment of adverse drug	Indications
reactions	Emphasis on reducing injections
**3- Drug Interactions (17, 18)**	Adverse effects
Important mechanisms of drug interactions	Important interactions
Common drug interactions in the practice of	B) Non steroidal anti inflammatory drugs
general physicians	(NSAIDs)
**4- Injections (20)**	Indications
Consideration of real needs for prescribing	Adverse effects
injections	Drug Interactions
Mechanism of injections	Contraindications
Indications for injections	
Important factors in prescribing injections	
Prevalence of prescribing injections in the	
world and in Iran	

* Numbers in parentheses refer to the related outcomes.

## Discussion

The Delphi technique was chosen in order to distil the opinions of several experts and reach consensus without geographical constraints [25] and with heterogenic groups [26]. A panel of selected experts has been used in other Delphi studies to identify prescribing errors in order to increase the validity of the findings [27,28]. We used a modified version with panel discussions and small working groups in order to better validate the results, minimize ambiguity, and further develop program content. The process yielded twenty-one outcomes and their related indicators. These included all but one of the initially proposed indicators and half of the additionally suggested ones (the indicator for name and age was divided into two separate ones to make it easier to assess). Based on discussions about the outcomes, six topic areas were identified and curricular content for each was determined by an expert team based on these outcomes. The content was then validated by all the participating experts.

We began the Delphi process by creating a preliminary list of potential learning outcomes through a literature review and then made use of experts' opinions to develop and prioritize these outcomes. These experts were involved in CME either as decision makers or trainers. GPs representing the target audience were also included as a way of improving the relevancy of the programme as has been described previously [9]. In these ways, we aimed to bridge the gap between the actual learning needs of individual practitioners and the educational content that is considered to meet assumed needs, a problem that has been described in the educational literature [29,30].

Two of the learning outcomes which were suggested by the experts during the Delphi process were excluded during the panel discussions. Due to a concern that the CME programme as a whole would be rejected if individual parts were considered to be too controversial, the outcome "writing main complaints of the patient in the prescription" was rejected. The panellists thought that it would be too great a departure from the current work practice of physicians. Even though the over prescribing of vitamins is thought to be a problem in Iran, the outcome, "appropriate number of vitamins", was rejected since it would be difficult to assess as most vitamins are not reimbursed by insurance organizations, the registries of which provide the best way to assess physician prescribing behaviour in Iran.

The panel added one outcome related to the prescribing of corticosteroids as it had been reported that corticosteroid drugs had been prescribed more than any other drug group in East Azerbaijan Province during the last year (yearly report of Rational Prescribing Committee, Tabriz University of Medical Sciences, Tabriz, Iran; 2005). The panel also emphasized the importance of the learning outcomes related to instructions on how to administer medications as there is evidence that there is a large number of prescriptions that do not include directions for patient use [16]. These were two examples, where outcomes from the original Delphi process were further modified or built upon with the modified Delphi process.

A comparison of the outcomes with the curricular content of the course that was developed reveals that while all of the outcomes are covered in the course, the entire course is not covered by the outcomes as the topic "adverse reactions to drugs" is not directly related to an outcome. ADR can be the result of irrational prescribing. Thus, knowledge about the consequences of irrational prescribing can motivate a change in participants' behaviour. As such, ADR can be considered integral to a course on rational prescribing. However, if the course was supposed to be designed using an OBE approach, why wasn't ADR identified as an outcome? Possible explanations are on the one hand that tradition prevailed and on the other that the specific construction of the task for the Delphi rounds had a decisive influence.

Regarding the first explanation, a review of the topics that were included in the curricula shows that these topics were taught in other CME courses on prescribing. Since the team of experts responsible for content development all had taught according to the earlier curricula, tradition might have prevailed despite receiving the finalized list of outcomes and an explanation about how they were derived.

The other more distinct and plausible explanation for why the initial Delphi process did not elicit a topic that content developers deemed essential could be due to the formulation of the instructions for the Delphi process. The task was to identify what should be included or considered when the doctor writes a prescription. We believe this to be the main reason for not suggesting ADR as an outcome indicator as information on ADR cannot be written directly in the prescription. However, since ADR has to be considered before a particular drug is chosen, content developers included this topic in the curriculum. While it would have been possible to develop direct outcomes and indicators for ADR, ADR can be seen to indirectly be part of and important in achieving some of the other outcomes, such as avoiding prescribing drugs with negative interactions or similar effects or prescribing injections too frequently.

A final observation was that the constructive alignment approach [31], where outcomes are tied together with content and assessment methods, was not expressly discussed during the third phase of content development. This could mean that content developers were simply unaware of the need to explicitly link outcomes to course content and could thus benefit from more knowledge about outcome-based education and constructive alignment prior to designing courses.

Another question concerns the quality of the outcomes and the indicators that were formulated. Outcomes can be analyzed based on how specific they are, if they are measurable, if they cover the domains of knowledge, skills, and attitudes, and if they are of a manageable number [32]. Most of the outcomes meet these criteria. However, the word "appropriate" (found in outcomes 15 and 19–21) is harder to measure, although it would be possible to measure if linked to clear guidelines. According to the ideas behind outcome-based education and constructive alignment, the validated indicators could form the basis for assessment of GPs' performance and provide them with feedback on the effectiveness of their learning. This could be pursued in further research.

## Conclusion

The modified Delphi process was effective in developing outcomes and indicators that were then used in the development of content for a course in rational prescribing. The indicators can be used to assess what participants have learned during the course as well as prescribing behaviour after the course. To improve the constructive alignment of the course, course developers should understand that outcomes need to be explicitly related to course curricula.

## Competing interests

The authors declare that they have no competing interests.

## Authors' contributions

HME: Study concept and design, collection of data, statistical analysis and interpretation of data, drafting of the manuscript and critical revision of the manuscript. CS: Study concept and design, interpretation of data, drafting of the manuscript and critical revision of the manuscript. RV: Study concept and design, collection of data, critical revision of the manuscript, acquisition of funding and study supervision. AA: Study concept and design, contribution to the manuscript, acquisition of data, acquisition of funding and study supervision. MHZ: Study concept and design, contribution to the manuscript. RW: Study concept and design, interpretation of data, critical revision of the manuscript and study supervision.

## Pre-publication history

The pre-publication history for this paper can be accessed here:


